# Perspective: Evolutionary Genetics Is the Backbone of Genomic Epidemiology in Fungal Pathogens

**DOI:** 10.3390/jof12100790

**Published:** 2026-10-21

**Authors:** Daniel R. Matute

**Affiliations:** Department of Biology, University of North Carolina, Chapel Hill, NC 27514, USA; dmatute@email.unc.edu

**Keywords:** phylogenetics, population genomics, molecular epidemiology, human fungal pathogens, genomic surveillance

## Abstract

Genome sequencing is rapidly becoming a routine component of outbreak investigation and pathogen surveillance. In human fungal pathogens, however, the analytical framework has often remained centered on a single tree built from variants called against one reference genome. While that representation has been and will continue to be useful, epidemiological inference can also benefit from other approaches drawn from evolutionary genetics. Here, I review the transition from multilocus sequence typing to genome-scale epidemiology in human fungal pathogens, summarize representative successes in *Coccidioides*, *Cryptococcus*, *Candida auris*, *Aspergillus fumigatus*, *Histoplasma*, *Sporothrix*, *Pneumocystis*, and other fungi, and identify biological and technical sources of error that are still inconsistently addressed. More than a critique, this piece is a call to action. I argue that we need to start treating phylogenetic trees as one layer of evidence within a broader population-genetic framework and not as a stopping point. From a data collection point of view, we need a more robust epidemiological metadata collection scheme. From an analytical point of view, analyses of gene-tree discordance, demographic inference, ancestry, and structural variation can reveal features that a single tree derived from concatenated markers cannot. The amount of public sequence data is no longer the only limiting factor. The next pressing challenge is to translate the data generated by genomic and phylogenetic analyses into reproducible evolutionary inference while preserving and communicating the uncertainty that matters for public-health decisions.

## 1. Introduction

The environmental conditions of our planet are changing at an unprecedented rate. Rising temperatures can alter the geographic distributions of pathogens and their hosts [1,2], change the seasonality of transmission [3,4], and reduce the thermal barrier that protects mammals from many environmental fungi [5,6,7,8]. Land-use change and deforestation increase contact among humans, domesticated animals, wildlife, and environmental reservoirs, while global travel allows an introduced pathogen to move farther and faster than at any earlier point in human history [9,10,11]. These changes alter exposure and change the ecological conditions that pathogens experience, the ways in which pathogens interact with their hosts, and ultimately their evolutionary trajectory. These ecological changes often leave signatures on the extent of genetic variation in the pathogen. Range expansions can produce founder effects and altered population structure, increased connectivity can change patterns of migration and gene flow, and new environmental or host-associated pressures can favor adaptive alleles, including those conferring antifungal resistance. When combined with environmental and epidemiological data, population-genomic analyses can help determine whether changes in disease occurrence reflect increased human exposure, changes in pathogen distribution and gene flow, or the emergence and spread of adaptive variants.

Understanding the processes that drive pathogen emergence and spread is critical for anticipating the trajectory of any disease. Genetic comparisons can reveal whether epidemics arise from single or repeated introductions and whether pathogens persist locally or arrive through recurrent migration. These comparisons can also reveal the contributions of clonal reproduction and recombination to pathogen diversity and track the spread of particular lineages. These are epidemiological questions, but they are also questions about ancestry, mutation, migration, and selection. Genetic epidemiology—and, when whole genomes are used, genomic epidemiology—therefore depends on evolutionary biology rather than simply co-opting its visual language [12]. Here, fungal genomic epidemiology is defined as the use of genome-scale variation, interpreted alongside clinical, environmental, and epidemiological information, to investigate the distribution, emergence, and spread of fungal disease. I include in its scope transmission reconstruction, population genomics and phylogeography, surveillance of antifungal resistance, within-host evolution, and environmental surveillance, thus linking evolutionary processes to patterns of infection and exposure.

Combining DNA sequencing with evolutionary analysis has improved our ability to track pathogen emergence and spread. In the case of HIV, sequence-based phylogenies have helped reconstruct the origins and geographic expansion of the pandemic [13,14,15,16] and identify rapidly growing transmission clusters [17]. When integrated with epidemiological investigation, cluster detection can guide the delivery of HIV testing, pre-exposure prophylaxis, and treatment services in affected communities [18,19]. A second notable example is that during the COVID-19 pandemic, the unprecedented release of SARS-CoV-2 genomes allowed public-health systems to detect new variants, estimate their growth and geographic spread, and evaluate changes in transmissibility in near real time [20,21,22,23]. However, genetic relatedness alone cannot establish direct transmission between two individuals because similar viruses may reflect infection from a common source or transmission through unsampled intermediates [24,25]. Together, these cases exemplify that genome sequencing is most informative when sampling is dense in time and space, the resulting genomes are linked to epidemiological metadata, and analyses explicitly model sequence evolution and the demographic processes relevant to the question.

The field has nevertheless not reached its full potential, rigor, or reach in eukaryotic pathogens. The study of fungal pathogens could benefit from the consistent application of the principles of genomic epidemiology. In this piece, I survey the status of genomic epidemiology in human fungal pathogens from the point of view of a population geneticist. I describe the transition from multilocus sequencing to genome-scale data, highlight what the field has already accomplished, identify biological and technical limitations that can mislead inference, and propose complementary approaches that can extract epidemiological information that is not contained in a single phylogenetic tree.

## 2. A Brief History of Genetic Epidemiology

Understanding adaptation and demographic history has always been one of the central tasks of evolutionary biology. As soon as sequence data became available, pathogen biologists adopted it to reconstruct disease events. DNA fingerprinting methods such as randomly amplified polymorphic DNA (RAPD) and amplified fragment length polymorphism (AFLP) enabled comparisons among fungal isolates without prior genome sequence information, advancing studies of population structure and potential epidemiological links (reviewed in [26,27]). These approaches provided valuable discriminatory power, but limited RAPD reproducibility complicated comparisons across laboratories. Moreover, fragments that migrated to the same position on a gel were not necessarily homologous, making shared banding patterns difficult to interpret as evidence of common ancestry and favoring the adoption of standardized sequence-based markers. Multilocus sequence typing (MLST), originally formalized for bacterial pathogens [28,29,30], provided a lifeline for population genetics and epidemiology by replacing morphology and laboratory-specific fingerprints with short, standardized DNA sequences that could be compared across studies and institutions [31,32]. A small set of PCR-amplified housekeeping loci could identify lineages, reveal geographic structure, and distinguish a persistent clone from a diverse collection of isolates.

The most common summary of these data has been a phylogenetic tree, a model of relationships in which branches represent ancestry and the accumulation of change. When calibrated with reliable sampling dates and/or mutation rates, trees can also inform the timing of divergence. Their appeal is obvious. A tree reduces a complex matrix of genetic differences to an intuitive representation that can be annotated with date, location, host, and drug susceptibility. That compression is powerful, but it also encourages the mistaken assumption that the evolutionary history of a locus, a concatenation of loci, a genome, and a lineage in a tree are interchangeable. They are not. Recombination, introgression, gene conversion, incomplete lineage sorting, and selection can cause different regions of a genome to have different genealogies. A handful of loci may therefore recover the dominant history, an unusual history, or simply an unresolved history. There is no universally ideal number of MLST loci [33], and how many loci are enough to identify instances of divergence will depend on the amount and distribution of polymorphism, recombination rate, population structure, divergence time, ploidy, and the epidemiological question to be answered. While simulations can estimate whether a particular marker panel separates the lineages and time scales relevant to a given study [34,35], no fixed number of unlinked loci guarantees an accurate species or transmission history.

Whole-genome sequencing greatly expanded the amount of genetic variation accessible to epidemiological analysis. The decline in sequencing costs, the maturation of short-read platforms, and the subsequent increase in long-read accuracy and read length have made entire fungal genomes accessible to population studies [36,37,38,39]. Genome-scale data allow for the study of SNPs and indels, haplotypes, copy-number changes, aneuploidy, structural variants, and within-host polymorphism in the same evolutionary framework (Box 1). Across the medically important fungal genera included in Figure 1, public whole-genome sequencing (WGS) runs released during the ten years from 2016 through 2025 contain 60.4 terabases of sequence. The cumulative total grew from 2.02 terabases at the end of 2016 to 60.4 terabases at the end of 2025. These figures are not counts of independent isolates—runs vary in depth, can be technical replicates, and include uneven taxonomic sampling—but they illustrate the magnitude of the analytical transition. Sequencing volume should therefore not be interpreted as equivalent to epidemiological representativeness. Even a large archive may disproportionately reflect particular outbreaks, geographic regions, or clinical samplings while leaving other populations and environmental reservoirs poorly sampled.

Box 1 **What whole-genome data add to genetic epidemiology.**

- Genome-wide substitutions and indels provide more markers for distinguishing closely related isolates than a small set of loci. For very short branches supported by few genetic differences, these data allow analyses to assess whether the inferred relationships are well supported or remain unresolved.
- Genome-wide data reveal combinations of variants inherited together as haplotypes. Their distribution along chromosomes can help identify regions inherited from different ancestral populations, locate recombination breakpoints, and detect patterns consistent with selective sweeps—features that isolated markers cannot adequately characterize.
- Differences in sequencing read depth can indicate gains or losses of genomic segments or whole chromosomes, revealing copy-number variation and aneuploidy. Comparisons among genome assemblies can identify genes present in some isolates but absent from others, as well as structural rearrangements such as inversions and translocations. These analyses capture variation that may be missed by SNP-based comparisons and can help investigate changes in gene dosage or gene content associated with antifungal resistance, virulence, and adaptation.
- Sequencing isolates collected from the same patient over time can reveal within-host genetic changes, including the emergence of resistance-associated variants during treatment. Sequencing multiple colonies from a sample or using deep sequencing can detect genetically distinct strains within an infection that a single isolate might miss. Comparisons between initial and recurrent infections can help distinguish relapse involving the original lineage from reinfection by a different lineage, although this distinction also requires clinical and epidemiological evidence.
- Sampling dates can calibrate molecular-clock analyses to estimate when lineages diverged, provided genetic change is sufficiently associated with elapsed time. Sampling locations support phylogeographic analyses of lineage distributions and movement among regions. When combined with appropriate models and epidemiological information, dated genomes can also support phylodynamic analyses of population growth and transmission, with uncertainty arising from incomplete or uneven sampling.
- Preserving raw sequencing reads allows variants to be recalled as reference genomes, gene annotations, and analytical methods improve. Sharing variant files, alignments, trees, and associated metadata also enables independent verification and reanalysis under alternative evolutionary models. Together with documented workflows, these resources allow existing datasets to address new questions and support comparisons across studies.

The combination of genome sequencing and phylogenetic analysis has already transformed viral and bacterial epidemiology [40,41,42,43]. This combined approach can identify pathogen introductions, distinguish local transmission from repeated importation, and track resistant lineages [44,45]. Additionally, the approach can assess whether outbreak cases are genetically consistent with a common source and identify possible gaps in transmission chains for further investigation [46,47]. Time-scaled and phylodynamic analyses can estimate lineage growth and migration, while the systematic incorporation of new sequences provides genetic surveillance and, in some instances, early warning of expanding lineages [42,48]. The take-home message of these developments is not that genome-based phylogenetic trees can replace epidemiological surveillance. On the contrary, genomic and population genetic data integrated with epidemiology can increase the resolution of the patterns of emergence of pathogens and the processes that lead to such emergence.

Eukaryotic pathogens have benefited less uniformly. Their genomes are generally larger than bacterial pathogen genomes, although the distributions overlap (Figure 2), and they commonly contain introns, repetitive DNA, transposable elements, subtelomeric gene families, variable ploidy, and multiple chromosomes. These genomic features complicate assembly, read mapping, phasing, and variant calling but are also important for understanding the biology of the pathogen. For example, ploidy changes are often related to the emergence of antifungal resistance [49,50]. The most significant limitation to the growth of genomic epidemiology in these pathogens, however, is not genome size. Fungal diseases are frequently underdiagnosed, many are not nationally reportable, and environmental reservoirs are sampled only sporadically [51,52,53]. For that reason, in many eukaryotic pathogens sampling is sparse. Sophisticated statistical and genomic tools cannot recover diversity that was never sampled, and a large sequence archive cannot supply the epidemiological denominator that was never recorded.

Malaria demonstrates that these obstacles are neither intrinsic to nor unavoidable in eukaryotic pathogens. Reference genomes and coordinated international sampling have made population genomics a practical component of *Plasmodium* research, allowing investigators to measure connectivity among parasite populations, distinguish local persistence from imported infection, and monitor the response to control programs [54,55,56]. Demographic analyses have revealed moderate rates of dispersal in *P. vivax* [57] and responses to selection imposed by antimalarials [58]. Genomic analyses also identified *kelch13* as a major determinant of artemisinin resistance [59,60], which in turn has made it possible to track resistant haplotypes across space and time [61,62,63]. Coordinated open resources now contain thousands of parasite genomes and standardized resistance markers, making it possible to move from retrospective description toward prospective surveillance [64].

## 3. Genetic and Genomic Epidemiology in Fungal Pathogens

Early multilocus studies established the promise of combining epidemiology and evolutionary biology in fungi. At deep and intermediate time scales, studies based on gene genealogies revealed geographically structured lineages within *Histoplasma* [65,66], *Paracoccidioides* [67,68], and *Coccidioides* [69,70]. Similar data indicated that *C. posadasii* expanded into South America, mirroring human migration patterns [71]. These studies showed that a morphologically defined pathogen could contain independently evolving lineages with different geographic ranges and, potentially, different clinical properties. Studies of fungal isolates collected from environmental sources also helped establish the foundations of fungal molecular epidemiology by revealing genetic diversity, geographic structure, and reproductive modes outside clinical settings [72,73,74]. For pathogens with environmental reservoirs, these studies provide the population context needed to interpret clinical isolates, investigate potential sources of exposure, and distinguish the distribution of infections from the distribution of the pathogen itself [75,76,77].

At shorter epidemiological scales, marker-based studies linked genotypes to outbreaks and transmission settings. PCR fingerprinting and amplified fragment length polymorphisms identified the rare *Cryptococcus gattii* genotype associated with the Vancouver Island outbreak and its environmental reservoir [78]. In *Candida*, standardized MLST schemes revealed major clades in *C. albicans*, geographic structure in *C. glabrata* and *C. tropicalis* [79,80,81,82], and evidence of clonality ([83,84] reviewed in [85]). These results also exposed a recurring limitation: a marker set that is excellent for global lineage assignment may contain too few mutations to resolve transmission within a ward, a household, or a short outbreak (reviewed in [86]).

Whole-genome studies have now answered questions that marker panels simply could not have addressed in the past. In *Coccidioides*, genome sequencing detected recent introgression between species [87,88], refined population structure across the Western Hemisphere [89,90], allowed clinical isolates from non-endemic locations to be connected to plausible regions of exposure [76,91,92], and uncovered drivers of genome evolution in the genus ([87,88,93] reviewed in [89]). Phylogeographic inference can help distinguish travel-associated infections from those acquired locally and identify regions where environmental sampling is most needed.

In *Cryptococcus*, WGS resolved the Pacific Northwest *C. gattii* outbreak into distinct sublineages, linked those sublineages to global diversity, and showed that clonal epidemic groups emerged from potentially recombining populations [94,95]. Genome-scale analyses of *C. neoformans* revealed global population structure, aneuploidy, candidate virulence-associated variants, and within-host changes during recurrent cryptococcal meningitis [96,97]. These studies illustrate two different—and complementary—axes of genomic epidemiology: reconstructing movement among populations and resolving evolutionary change within an individual infection.

*Candida auris* provides one of the clearest examples of fungal genomics entering public health practice. WGS demonstrated that deeply separated clades emerged nearly simultaneously on different continents, while dense hospital sampling resolved local transmission, environmental persistence, and repeated introductions [98,99,100]. The global emergence of *Candida auris* as a multidrug-resistant hospital pathogen highlights the importance of understanding the origins and spread of resistance [101]. At the cellular level, gene duplication has been associated with increased fluconazole tolerance in cells of advanced generational age [102]. At the population level, a global analysis of 304 isolates documented geographic spread within genetically distinct clades, estimated recent clade expansions, and identified differences in resistance-associated variants among clades [103]. Interpreting these patterns requires an understanding of their evolutionary origin. Clade-associated resistance does not necessarily imply a single evolutionary origin. Resistance may spread through the expansion of a resistant lineage, arise independently in different genetic backgrounds, or be acquired through gene exchange. Distinguishing these histories requires examining resistance alleles and their surrounding haplotypes, connecting surveillance to the problem of molecular convergence discussed below.

Studies of other fungal pathogens also combine genomic and environmental data to investigate sources of infection. Clinical and environmental genomes support environmental acquisition of azole-resistant *Aspergillus fumigatus* [104]; WGS showed that the post-tornado cluster of *Apophysomyces* mucormycosis involved multiple strains rather than one clone [105]; and population genomics has informed our understanding of the zoonotic spread of *Sporothrix brasiliensis* [106] and genotype-phenotype associations in *Talaromyces marneffei* [107].

A separate question concerns the genomic mechanism underlying resistance. In addition to nucleotide substitutions, copy-number changes, aneuploidy, and other structural variants can alter the dosage of antifungal targets and regulators of drug efflux. Genome-wide analyses of *Candida albicans* have identified repeat-associated rearrangements that generate copy-number variation and loss of heterozygosity, revealing a source of variation that SNP-based analyses alone cannot fully capture [108]. These changes can have direct functional consequences: amplification of ERG11 and TAC1 through formation of a chromosome 5 isochromosome increases fluconazole resistance, while expandable and reversible segmental amplifications can facilitate rapid adaptation to antifungal exposure [109,110]. Incorporating structural-variant screening into genomic surveillance can therefore help explain differences in susceptibility among closely related isolates and identify resistance mechanisms overlooked by analyses restricted to nucleotide substitutions.

Public sequencing data reflect the growing use of genomics in fungal epidemiology, but coverage remains uneven across pathogens. For the ten pathogen-rich eukaryotic genera in Figure 3, WGS run data released from 2016 through 2025 total 512 terabases. The fungal genera account for 9.7% of those bases and the protozoan genera for the remaining 90.3%. Within the fungal panel, *Cryptococcus* accounts for 11.9 terabases across the decade, whereas substantially fewer sequencing data are available for several endemic dimorphic fungi such as *Coccidioides*. Although coverage remains uneven across pathogens, regions, and clinical syndromes, publicly available WGS data now extend beyond individual reference genomes to population-scale collections, providing a broader basis for studying pathogen evolutionary history.

The limiting factor is consequently shifting. For some fungi, generating a genome remains difficult because the organism cannot be cultured, the pathogen burden in a specimen is low, or host DNA dominates. In some instances, these three factors contribute to the difficulty of studying the pathogen (e.g., *Paracoccidioides lobogeorgii*, [111]). For others, thousands of runs already exist and the challenge is data harmonization: identifying independent isolates, recovering sampling metadata, reconciling taxonomic names, and standardizing read mapping and variant calling. Large sequencing datasets become epidemiologically useful only when their metadata and sampling frame allow the underlying biological question to be defined.

## 4. Current Approaches

Fungal genomic epidemiology studies commonly summarize genetic relationships using a single tree or genomic distance measure, while fewer explicitly account for the evolutionary processes that complicate these relationships. Table 1 compiles 30 representative fungal genetic and genomic epidemiology examples spanning species delimitation, regional dispersal, hospital outbreaks, zoonotic epidemics, and within-host evolution. The compilation is meant to serve as a sample and is not a complete review, so the counts should be interpreted descriptively. Thirteen entries summarize primarily marker-based approaches, and 17 summarize genome-scale approaches. These categories are not mutually exclusive: the *Sporothrix* entry includes both targeted-marker and genome-based studies. Among the WGS entries, 15 ultimately summarize relatedness with a single concatenated SNP tree or pairwise genomic distance representation, and most begin with variants called after short reads are mapped to one linear reference. Only a minority explicitly model recombination, gene-tree discordance, or uncertainty in divergence time. Here, I review approaches for inferring phylogenetic relationships and evaluating how consistently those relationships are supported across the genome.

### 4.1. Reference-Mapped SNP Phylogenies

#### 4.1.1. Maximum-Likelihood Inference

A common workflow in fungal pathogen population genetics involves mapping reads to a reference genome, calling variants, and filtering sites by depth and quality. The retained SNPs are then concatenated and used to estimate a maximum-likelihood tree, whose topology summarizes relationships among isolates and whose branch lengths represent estimated genetic divergence. RAxML [129] and IQ-TREE [130,131] are commonly used for this approach. This pipeline is computationally efficient and can resolve relationships among closely related haploid isolates. Reproducibility requires reporting the reference genome, variant-calling procedures, filtering criteria, and phylogenetic settings. Several choices can affect the resulting tree, including divergence from the reference, treatment of heterozygous sites, assumptions about ploidy, masking of repetitive regions, and treatment of invariant callable sites when estimating branch lengths.

#### 4.1.2. Bayesian Inference

Bayesian phylogenetic methods estimate trees while integrating uncertainty in topology and model parameters. MrBayes [132] is commonly used for phylogenetic inference, whereas BEAST [133,134] supports estimation of dated phylogenies and demographic histories under molecular-clock and population models [135]. These methods can analyze reference-derived SNP alignments, although neither Bayesian nor maximum-likelihood inference inherently requires mapping reads to a reference genome. Studies using Markov chain Monte Carlo should report the priors, chain lengths, burn-in, effective sample sizes, and evidence of convergence across independent runs. Calendar-time inference also requires appropriate calibration. Fungal datasets may lack sufficient temporal signal over the sampling interval, and limited knowledge of mutation rates and generation times can further constrain estimates of epidemiologically relevant dates.

#### 4.1.3. Gene-Tree and Species-Tree Inference

Different genomic regions can have different evolutionary histories, so a tree estimated from concatenated sites may not capture all relationships present in the genome. Gene trees can be inferred for individual loci using maximum-likelihood or Bayesian methods, and species-tree methods then combine information across those genealogies. ASTRAL, for example, estimates a species tree from gene trees under the multispecies coalescent framework [136,137]. These approaches accommodate discordance associated with incomplete lineage sorting, but their interpretation depends on the quality of the input gene trees and the assumptions of the model. Recombination within loci, gene flow among lineages, and limited variation in individual genes can complicate inference. Species-tree methods therefore complement concatenation, particularly when the goal is to resolve relationships among divergent lineages rather than closely related outbreak isolates.

#### 4.1.4. Branch Support and Genomic Concordance

Support statistics help assess confidence in inferred relationships, but different measures quantify different aspects of uncertainty. These measures apply to trees inferred from a variety of sequence alignments, regardless of whether the data were generated by mapping reads to a reference genome. Standard bootstrap support is the proportion of replicate analyses that recover a clade after alignment sites are resampled with replacement; it is not the probability that the clade is true [138]. Ultrafast bootstrap provides a computationally efficient approximation to bootstrap analysis [139]. The SH-like approximate likelihood-ratio test (SH-aLRT) evaluates support for a branch relative to local alternative arrangements [140]. Bayesian posterior probabilities describe the probability of a clade conditional on the observed data, evolutionary model, and specified priors. These values should not be interpreted interchangeably. Moreover, high support can coexist with systematic error: large SNP datasets do not eliminate reference bias or model misspecification, and standard site resampling does not account for linkage among sites.

Concordance factors address a complementary question: how consistently different parts of the dataset support a branch. The gene concordance factor is the percentage of decisive gene trees containing that branch, whereas the site concordance factor summarizes the proportion of decisive sites supporting it across sampled quartets of taxa [141]. A branch can therefore have high bootstrap support but low concordance, indicating that a strongly supported concatenated tree may not represent a history shared across the genome. Low concordance can reflect biological discordance or limited phylogenetic information and does not, by itself, establish recombination or introgression.

Support statistics should be interpreted alongside branch lengths, genomic concordance, sensitivity to masking and recombination, and alternative topologies. Weakly supported branches identify relationships whose epidemiological interpretation remains uncertain and should be reported as such.

### 4.2. Alignment- and Reference-Free Approaches

The potential pitfalls of using a reference genome have been described before and solutions to these limitations have also been proposed [142,143,144]. Reference-free approaches compare k-mers derived from reads or assemblies, avoiding dependence on read mapping to a single reference genome. kSNP identifies SNPs without a reference alignment, whereas Mash uses MinHash sketches to estimate genome distance rapidly [145,146]. A variety of alternatives with the same premise have been designed to infer the evolutionary history of bacterial lineages [147,148,149]. The advantage of these tools is their reduced dependence on a single reference genome, potentially reducing alignment and variant call biases. As with any other method, there are limitations. k-mer distances combine substitutions, gene-content differences, contamination, assembly quality, repetitive DNA, and sequencing error. Only a few studies in fungal epidemiology have used these approaches to infer genealogical relationships among isolates (e.g., [150,151,152,153]). Even this low number of implementations suggests the approach is feasible.

### 4.3. Variation in Genealogies Across the Genome

Beyond estimating a summary tree, analyses can examine how genealogical relationships vary across the genome. Trees inferred for individual genes or genomic windows identify regions supporting alternative relationships, while topology weighting quantifies the relative representation of specified topologies along the genome [154]. These approaches complement the species-tree and concordance analyses described above by locating discordance and characterizing its genomic distribution. In fungal epidemiology, they can help identify regions with histories consistent with gene exchange and explain why whole-genome trees inferred from different marker sets disagree. Interpretation requires accounting for incomplete lineage sorting, variation in phylogenetic information, and technical artifacts; alternative topologies alone do not establish recombination or introgression.

## 5. Limitations and Solutions

The integration of genomic data into phylogenetic reconstruction has introduced challenges that were neither encountered—nor could have been anticipated—by MLST-era workflows. Some represent biological phenomena and therefore contain information about pathogen evolution. Others arise from bioinformatic choices and can produce patterns that misrepresent the evolutionary history of the organism. The distinction matters. Biological discordance should be modeled and dissected because it reveals features of the pathogen’s evolution, whereas technical discordance should be diagnosed and minimized.

### 5.1. Biological Challenges

Gene exchange and introgression: Hybridization can create a bridge for gene transfer when hybrids or their descendants reproduce [155,156]. Introgressed segments may show different tree topologies, grouping the exchanging lineages more closely than non-introgressed regions, which usually comprise most of the genome. Recent introgression can also reduce sequence divergence between those lineages, producing shorter genetic distances and more recent inferred coalescence times in the affected regions. In fungi, hybridization and introgression have contributed to the origin of pathogenic lineages, ecological shifts, and drug resistance; *Candida* provides a direct example in the genomic-epidemiological literature [157,158,159,160,161]. No single method is sufficient to detect the relative importance of introgression across every time scale, and corroboration across methods strengthens the inference. The battery of tools for detecting introgression is vast, but among the most powerful and widely used are *D*-statistics [162,163] and their derivatives [164,165,166], which test asymmetry in allele sharing; topology weighting, which localizes alternative genealogies [154]; phylogenetic networks, which represent reticulate histories [167,168]; and ancestry or haplotype methods, which can identify recently admixed genomic tracts [169,170,171,172]. The first three are powerful for detecting older admixture events, whereas the latter are best suited to recent events.

Incomplete lineage sorting: Incomplete lineage sorting (ILS) occurs when ancestral polymorphism persists across successive speciation events. In these instances, the resulting gene trees can differ from the population or species tree. Compared with recent introgression, discordance caused by ILS is generally expected to occur in shorter genomic segments because ancestral haplotypes have had more time to be broken up by recombination. Under ILS alone, coalescence between lineages from different species predates their divergence [173,174,175]. This simple expectation is complicated by selection, recombination-rate variation, and effective population size [34,176,177]. Although ILS might seem like an obscure phenomenon with little relevance for genomic epidemiology, it is particularly common among recently diverged lineages—which are often the focus of epidemiological studies—and can therefore affect inferred relationships. The integrative use of multispecies-coalescent methods and simulations under fitted demographic models provides a strong basis for distinguishing introgression from ILS [175,178].

Convergence in molecular sites: Identical molecular variants can have different evolutionary origins. A small number of homoplastic sites may have little effect on a well-resolved genome-wide tree, but their influence can be substantial when relationships depend on only a few informative variants. Surveillance programs, however, must account for this possibility because a variant of concern may arise independently multiple times. The genome-wide contribution of molecular convergence remains poorly characterized in pathogenic fungi. When a variant is potentially associated with a phenotype of public-health importance, such as antifungal resistance, it is worth inferring the history of the resistance allele and that of its surrounding haplotype. The distinction between one successful resistant clone and repeated evolution of resistance is, in and of itself, a central epidemiological result.

### 5.2. Technical and Inferential Artifacts

Tree rooting and presentation: An unrooted tree contains evolutionary information about splits and genetic distances. Nonetheless, without a root, the tree does not specify the direction of evolution or establish the relative order of divergence events. One alternative is midpoint rooting. Although this approach may seem arbitrary, it has an explicit assumption: evolution is approximately clock-like. The method places the root at the midpoint of the longest path between two tips. However, the clock-like assumption is often untested, and midpoint rooting is sensitive to variation in evolutionary rates among lineages and to long branches. A closely related outgroup that has not exchanged genes with the ingroup (i.e., the focal study group) is generally preferable when available. Every tree should also state how it was rooted—or explicitly indicate that it is unrooted—include a branch-length scale and its units, identify collapsed clades, and make clear that rotating nodes does not alter the topology.

Insufficient resolution at the relevant time scale: Recent divergences are highly important for epidemiological studies and are likely to be separated by only a few mutations. Conserved MLST loci, ribosomal markers, and ultraconserved elements may assign a lineage correctly while containing no information about a local outbreak. Whole-genome sequencing increases the number of observable sites, and thus the power to detect recent divergences, but more molecular markers do not guarantee genealogical resolution when mutation rates are low, sampling intervals are short, or a clone has expanded rapidly. Before drawing transmission conclusions, studies need to assess the expected number of mutations over the sampling interval—that is, whether there is sufficient power to detect divergence.

Reference mapping and reference bias: Mapping probability decreases as a read diverges from the reference [144,179,180]. Thus, using a highly divergent reference genome might lead to underestimating variants and thereby affect downstream analyses [143,144]. This problem has been observed, for example, in *Paracoccidioides* [181]. In the case of phylogenetic trees, the resulting missingness is nonrandom and might influence the topology of the tree and branch lengths. Phylogenetic inference in *Blastomyces* shows that the choice of reference genome affects both tree topology and branch lengths [153]. Repeating the analysis using references from different clades is a useful diagnostic, but the approach is ultimately limited because it cannot establish which inferred tree is correct. *De novo* assembly and pangenome graphs (i.e., graphical representations of genomic variation across multiple individuals as interconnected alternative sequences rather than as deviations from a single linear reference genome, [182]) provide stronger alternatives when gene content and structural variation matter [183,184,185,186]. An assessment of the impact of reference choice in fungal genomics is sorely needed.

Comparability and reproducibility: Until recently, generating whole-genome data for population-genetic and epidemiological analyses was prohibitively expensive. A less expensive alternative has been to obtain marker data from only a portion of the genome. The question then becomes which portion to sequence. Reduced-representation methods—those that sequence only part of the genome—can generate thousands of markers simultaneously, providing substantial information for a focal study. The problem is that the same loci are not always recovered across studies or divergent populations. Restriction-site polymorphisms in ddRAD-seq, for example, cause allele dropout and make comparisons dependent on the restriction enzymes, size-selection criteria, and library-construction methods used [187], potentially rendering comparisons across studies impractical, if not impossible. Although WGS datasets are not automatically comparable, the challenges affecting WGS studies—including reference-genome choice, ploidy assumptions, filtering criteria, and the treatment of repeats and heterozygosity—are compounded in reduced-representation studies by the additional problem of inconsistent locus recovery.

Sampling, metadata, mixed infection, and ascertainment: Phylogenetic reconstruction is subject to the same limitations as any experimental endeavor and depends heavily on the quality of the sampling design. Oversampling an outbreak clone can distort demographic inference by misrepresenting allele frequencies. Consensus-genotype calling, particularly when sequencing coverage is low, can also obscure mixed infections and within-host variation. Currently, few standards exist for addressing these potential issues. Ideally, prospective studies should clearly define and report the source population and sampling fraction, even if the latter can only be estimated. Most important, however, are the collection, preservation, and reporting of metadata. Sampling dates and locations, specimen types, treatment histories, patient travel histories, and environmental links should be recorded using controlled vocabularies. These metadata are essential for interpreting divergence among isolates and its public-health significance. Genetic proximity can help narrow an epidemiological investigation, but it cannot, by itself, establish direct person-to-person transmission or its direction.

Phylogenetic method, recombination, and genome state: Different tree construction algorithms often recover the same major groups, but not always [153]. Technical decisions—such as the treatment of near-zero-length branches, missing data, invariant sites, and recombination—can alter both topology and branch lengths. To complicate matters, fungi have plastic genomes that a SNP matrix based on a single ploidy level may not represent appropriately. Loss of heterozygosity, aneuploidy, segmental duplication, and parasexual recombination are all biological realities that a fixed ploidy model disregards. Sensitivity analyses should compare reasonable substitution models and inference methods, analyze recombination-masked and unmasked datasets, and report whether copy number and heterozygosity support the SNP-tree groups.

Branch lengths and the timing of divergence: Branch lengths quantify inferred evolutionary change and can inform divergence times when interpreted under an appropriately calibrated molecular-clock model. A branch measured in substitutions per site is not automatically a calendar interval, and a branch measured in SNPs depends on biological features and the reliability of variant calling. Recombination can increase or decrease local genetic distances between isolates, depending on the ancestry of the exchanged segments. Purifying selection removes variants, and latency or environmental growth decouples elapsed time from cell division. Moreover, pathogen trees can rarely be calibrated with fossil records. Time-scaled inference should demonstrate temporal signal with date-randomization analyses, compare different models of molecular evolution (e.g., strict and relaxed clocks), be cognizant of uncertainty in mutation rates, and state whether the estimated date refers to a sampled common ancestor, a population split, or a transmission event. Those quantities are related but are not identical.

## 6. Alternative and Complementary Approaches

Phylogenetic trees are indispensable for current epidemiological approaches, but they are not the only evolutionary tool that can inform an outbreak or, more generally, reveal how pathogen lineages are related. Here I present five complementary approaches that could also be informative for epidemiological inference.

### 6.1. Demographic Models and Divergence Time

Site-frequency-spectrum methods such as dadi [188,189], GADMA [190], and fastsimcoal [191] can compare explicit histories containing population splits, bottlenecks, growth, migration, and admixture and estimate their parameters. They are particularly useful when the question is whether an epidemic lineage arose through a severe bottleneck, whether two regions exchange migrants, or whether divergence continued in the presence of gene flow. They also compare observed data with null hypotheses, providing a benchmark. Novel machine-learning approaches [192,193] offer new tools to infer demographic patterns and quantify important evolutionary parameters such as recombination landscapes [194] and migration rates between populations [195].

### 6.2. Changes in Effective Population Size

Sequentially Markovian coalescent methods—including PSMC [196], MSMC [197], and SMC++ [198]—use the distribution of coalescent times along the genome to infer changes in the historical effective population size (*N*_e_), which governs the rates of genetic drift and coalescence under the assumed population model. Note that their applicability depends on ploidy, sampling, and recombination, and their resolution may be insufficient for demographic changes occurring over the duration of a recent outbreak. Machine-learning methods provide a complementary approach. For example, donni uses neural networks trained on simulated allele-frequency spectra to estimate demographic parameters, including historical population-size changes, and their uncertainty [199]. An outbreak founded by a small number of genotypes may have a genetic signature of an initial bottleneck followed by population expansion. The distinction between *N*_e_ and census size is critical for the interpretation of results obtained with these approaches. Case counts may rise rapidly while *N*_e_ remains small. Population structure, selection, clonality, and changes in recombination can also produce signals that resemble changes in population size. These methods are best viewed as models of coalescent history whose epidemiological meaning must be tested against sampling dates and case counts.

### 6.3. Clustering and Ancestry

A different family of methods does not aim to estimate genealogical relationships among individuals but instead aims to establish how many clusters best explain the allele frequencies observed in a dataset. STRUCTURE [200], fastSTRUCTURE [201], and ADMIXTURE [202] estimate assignments to genetic clusters under explicit population models without forcing a bifurcating history. These approaches complement a tree by estimating the most likely number of clusters and identifying samples that do not fit neatly in discrete clades. They do not, on their own, demonstrate admixture. Fractional cluster assignments can reflect gene flow, shared ancestral variation, continuous population structure, uneven sampling, or departures from the clustering model. Distinguishing among these explanations requires combining clustering results with evidence from local genealogies, allele-sharing tests, and explicit demographic models.

### 6.4. Phylodynamics and Transmission Models

Properly dated trees can be linked to birth–death, coalescent, or structured models to estimate lineage growth, migration, and changes in transmission. Tools such as TransPhylo [47] and outbreaker2 [203] combine pathogen genomes with sampling times and epidemiological information while allowing for uncertainty due to unsampled cases. These models are attractive for hospital outbreaks, but their resolution depends on the amount of informative genetic change accumulated over the sampling interval. Outbreak isolates may be indistinguishable at the analyzed sites, leaving their relative branching order unresolved. In such instances, patient clinical history and environmental sampling may contain more information about transmission than another tree-building algorithm.

### 6.5. Pangenomes and Structural Variation

Reference genomes are often selected for historical or practical reasons and may not adequately represent the diversity of the populations under study. Moreover, linear reference genomes represent an incomplete model when there are accessory genes, inversions, translocations, copy number variants, and aneuploidy. All these types of variants exist in fungi and can contribute to virulence or resistance. Multiple high-quality assemblies and pangenome graphs can represent a sequence that is absent from the canonical reference and make structural differences comparable across isolates without giving more weight to a reference genome chosen arbitrarily (discussed above). By capturing and representing accessory genes and variation in genome structure, pangenomic analyses provide a reproducible framework for identifying determinants of antifungal resistance, virulence, and adaptation to host and environmental conditions. In *Aspergillus fumigatus*, for example, these approaches have already identified accessory genes associated with azole resistance and clinical origin, and revealed lineage-specific metabolic gene repertoires consistent with environmental niche differentiation [104,204,205]. As is the case for all genomic surveys, these findings identify candidates for functional testing rather than, by themselves, demonstrating that particular accessory genes cause resistance or adaptation. Nonetheless, this integration has the potential to change epidemiological questions from ‘which SNP-defined lineage is this?’ to ‘which combination of alleles, genes, and chromosome states is moving through the population?’

## 7. Conclusions

Genomic epidemiology has accrued enough successes to earn a place in the toolkit of both epidemiologists and evolutionary geneticists. The amount of available fungal genome data has also exploded, and it is time to realize the potential of combining genomics, phylogenetics, and epidemiological principles to answer pressing questions about transmission clusters, the spread of antifungal resistance, and the relative importance of environmental acquisition. The field will become more useful as sequencing moves closer to routine public-health surveillance and as sampling expands beyond a small number of pathogens and countries.

More data—both in the form of sequenced isolates and of genetic markers—do not make assumptions disappear. A single concatenated tree can be an excellent summary of a clonal outbreak and a poor summary of a recombining or structured population, or of one undergoing introgression. Reference bias, uneven sampling, mixed infections, and uncertainty about the clock-like behavior of molecular evolution can produce apparently precise estimates that are nevertheless biased or misleading. The solution is not to abandon trees but to integrate them with other approaches that compare local genealogies, demographic histories, ancestry, genome structure, phenotype, and epidemiological evidence. For a phylogenetic tree to be reusable, studies should release raw reads, sample metadata, the final variant set and mask, trees in Newick or Nexus format [206], code or a versioned workflow, and the identity of the reference genome. Repositories such as TreeBASE, Dryad, Zenodo, and the Open Tree of Life can preserve and disseminate phylogenetic results. The FAIR principles—findability, accessibility, interoperability, and reuse—are not just lofty goals to be achieved in the future [207]. Instead, their application should be a prerequisite for cumulative evolutionary epidemiology.

My argument is straightforward. Both evolutionary and epidemiological principles need to inform how trees are built. Evolution is the guiding principle behind the tree. Epidemiology translates the tree into the current biology of the pathogen. For evolutionary genetics to remain applicable and build on previous studies, an old challenge remains: we need to maintain and distribute isolate metadata along with the published results. The time might be ripe for a proper centralized repository storing isolate metadata. Fungal genomic epidemiology will reach its potential when it leverages the power of this evolutionary framework with the same care devoted to generating the sequence itself.

Ultimately, fungal genomic epidemiology should integrate genomic, clinical, environmental, and epidemiological evidence to strengthen, rather than replace, conventional epidemiological investigation.

## Figures and Tables

**Figure 1:**
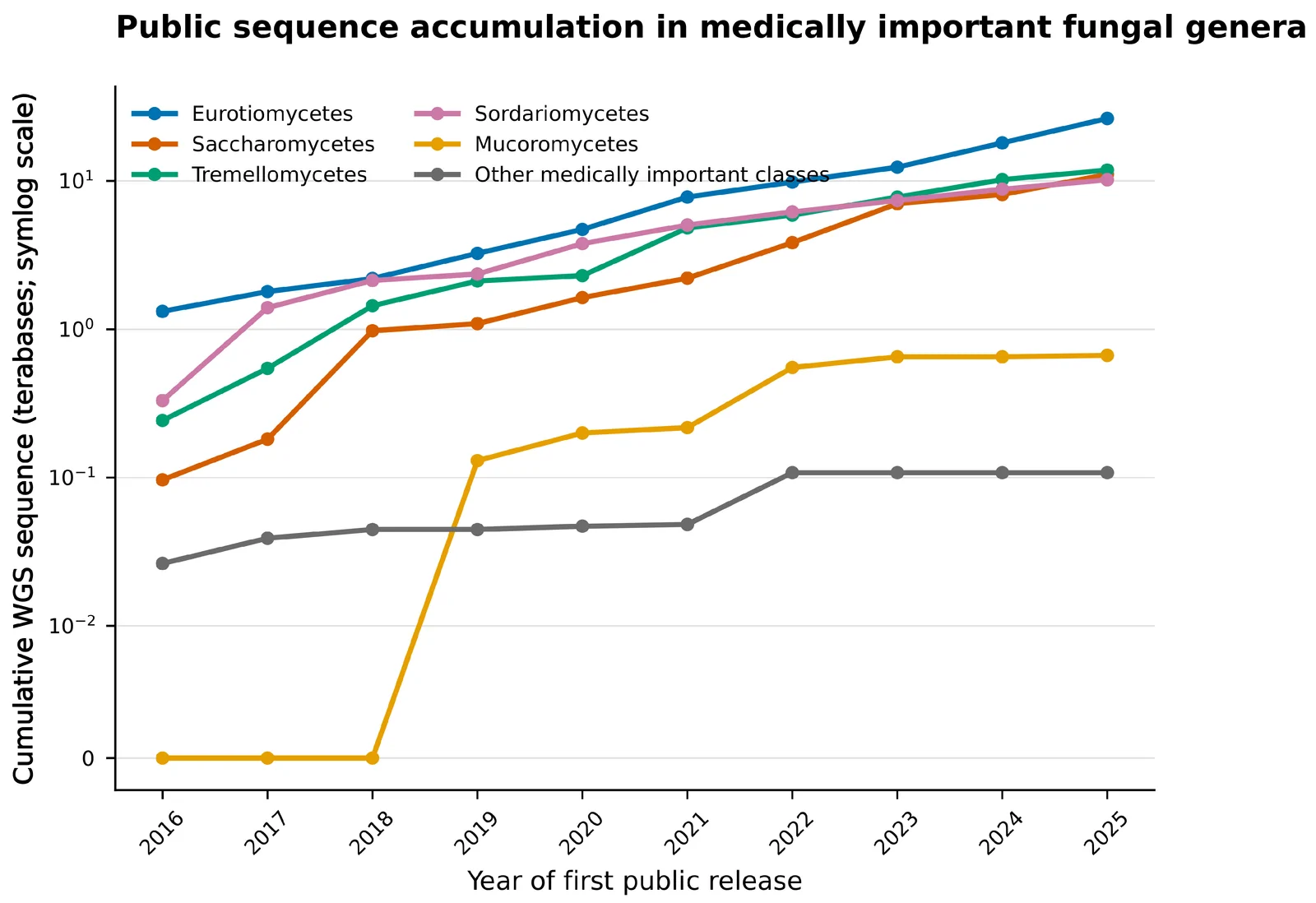
Accumulation of public whole-genome sequence data for medically important fungal genera, grouped by fungal class, 2016–2025. Curves show cumulative terabases in public WGS runs with library source GENOMIC and first-public dates in each complete calendar year. Classes are represented by selected genera listed in the source CSV; the figure is therefore an index of public-data growth, not a census of all human-pathogenic fungi or of independent isolates. Run metadata were retrieved on 30 July 2026 through the ENA Portal API, which mirrors public INSDC/SRA records.

**Figure 2:**
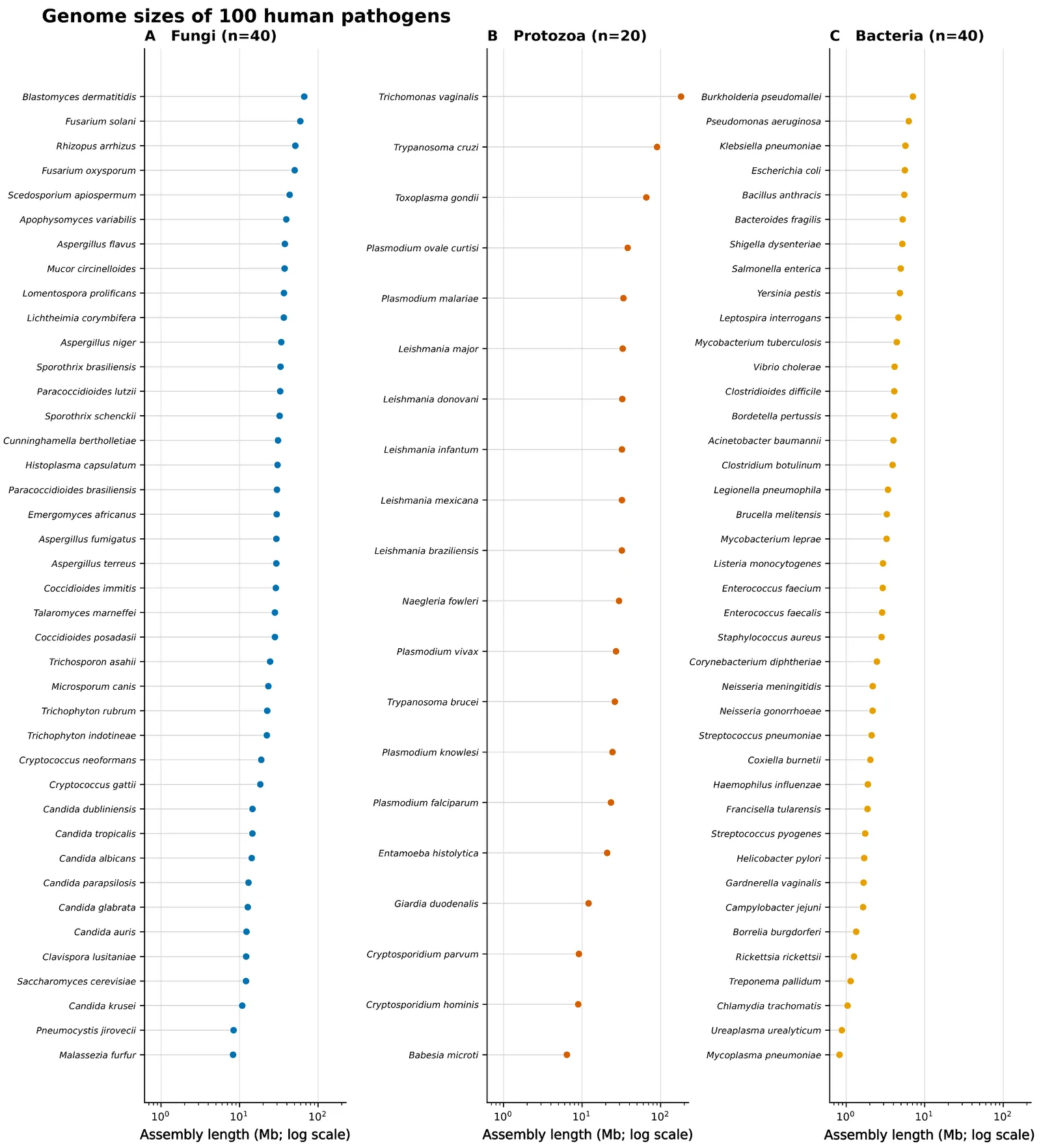
Reference or representative genome sizes for 100 human pathogens. Points show assembly length in megabases on a logarithmic axis for 40 fungal pathogens (blue), 20 protozoan pathogens (red), and 40 bacterial pathogens (yellow). When an NCBI reference assembly was unavailable, a current RefSeq or highest-quality available assembly was selected according to assembly level. Values describe the displayed assembly rather than an invariant species-wide genome size and can be affected by ploidy representation, haplotype separation, unresolved repeats, and assembly completeness. NCBI Datasets was accessed on 30 July 2026.

**Figure 3:**
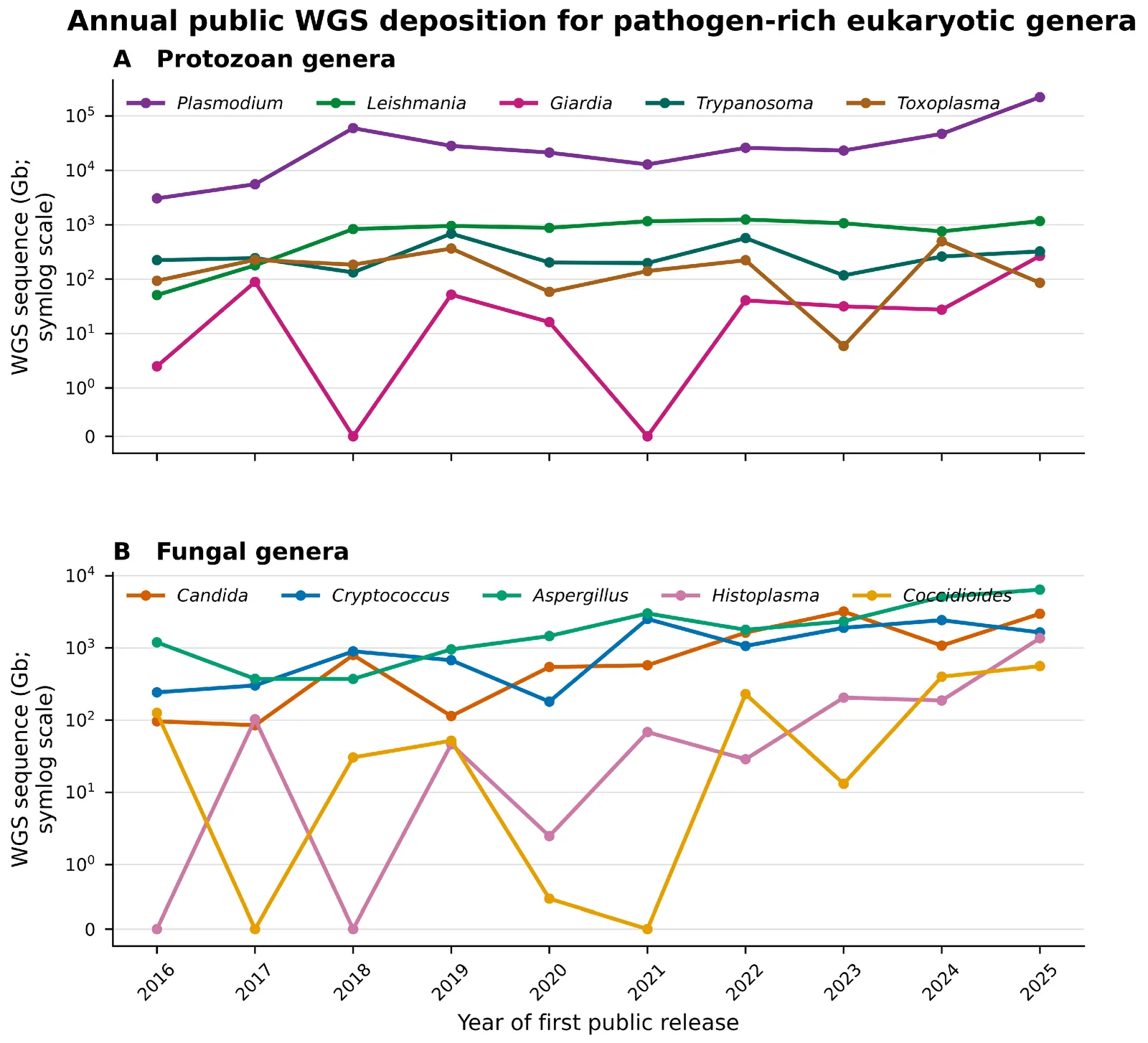
Annual deposition of public whole-genome sequence data for ten pathogen-rich eukaryotic genera, 2016–2025. The upper panel shows five protozoan genera (*Plasmodium*, *Leishmania*, *Giardia*, *Trypanosoma*, and *Toxoplasma*); the lower panel shows five fungal genera (*Candida*, *Cryptococcus*, *Aspergillus*, *Histoplasma*, and *Coccidioides*). Points represent gigabases in public WGS runs with library source GENOMIC and a first-public date in the indicated year; the y-axis is logarithmic. Genus-level queries include pathogenic and nonpathogenic members where both exist and should not be interpreted as disease burden or numbers of independent clinical isolates. Run metadata were retrieved on 30 July 2026 through the ENA Portal API mirror of public INSDC/SRA records.

**Table 1:** Representative examples using multilocus or whole-genome data to investigate the evolutionary genetics and epidemiology of human fungal pathogens. Studies are organized by the principal temporal scale of inference. The compilation is illustrative rather than a systematic review.

Temporal Scale	Taxon	Scope and Main Result	Primary Approach	Reference
Deep history/species boundaries	*Histoplasma capsulatum* species complex	Global phylogeography. Resolved geographically structured lineages and showed that the traditional varieties concealed multiple phylogenetic species.	Four unlinked protein-coding loci; genealogical concordance; distance and parsimony trees	[66]
Deep history/species boundaries	*Paracoccidioides brasiliensis* species complex	Species delimitation across Latin America. Identified three reproductively isolated phylogenetic species and evidence of recombination within one lineage.	Eight nuclear loci; genealogical concordance; recombination tests	[67]
Deep history/species boundaries	*Coccidioides immitis/C. posadasii*	Expansion into South America. Supported a recent expansion of *C. posadasii* into South America that broadly mirrored human movement into the continent.	Multilocus sequence data plus microsatellite genotypes; phylogeography and coalescent inference	[71]
Deep history/species boundaries	*Blastomyces dermatitidis* complex	Cryptic species discovery. Identified *B. gilchristii* as a phylogenetically distinct species within isolates previously called *B. dermatitidis*.	Seven-locus multilocus sequence analysis; concatenated and concordance phylogenies	[112,113]
Deep history/species boundaries	*Cryptococcus neoformans/C. gattii* species complexes	Lineage delimitation. Recovered six major monophyletic lineages and clarified relationships among molecular types.	Multilocus sequence typing; concatenated maximum-likelihood and Bayesian phylogenies	[114]
Deep history/species boundaries	*Sporothrix pathogenic* clade	Species relationships and geographic structure. Separated the principal agents of sporotrichosis and linked major lineages to geographic and transmission patterns.	Targeted-marker sequencing and whole-genome phylogenetics. ITS, calmodulin and beta-tubulin sequencing; concatenated phylogeny from genome sequences	[115,116]
Contemporary/outbreak scale	*Cryptococcus gattii*	Vancouver Island outbreak. Identified a rare VGII genotype shared by clinical and environmental isolates during the Vancouver Island outbreak.	PCR fingerprinting and amplified fragment length polymorphism typing	[78]
Contemporary/population scale	*Candida albicans*	Global population structure. Delineated four major and eight minor clades and demonstrated the portability of fungal MLST for large isolate collections.	Seven-locus MLST; clustering and distance-based analysis	[82]
Contemporary/population scale	*Candida glabrata*	Global clinical diversity. Defined geographically enriched clades while retaining evidence compatible with occasional recombination.	Six-locus MLST; sequence-type clustering, linkage disequilibrium and phylogenetic compatibility analyses.	[79,117]
Contemporary/population scale	*Candida tropicalis*	Clinical strain differentiation. Established a six-locus typing scheme with substantially greater discrimination than single-marker approaches.	Six-locus MLST; sequence-type and cluster analysis	[81]
Contemporary/population scale	*Candida dubliniensis*	Global clinical diversity. Revealed a predominantly clonal population with a small number of deeply separated clades.	Multilocus sequence typing; cluster and phylogenetic analysis	[118]
Contemporary/outbreak scale	*Pneumocystis jirovecii*	Liver transplant unit outbreak. Genotyping supported a common source or transmission chain among epidemiologically linked cases.	Multilocus sequence typing integrated with patient-contact investigation	[119]
Contemporary/outbreak scale	*Pneumocystis jirovecii*	Heart transplant clinic outbreak. A shared multilocus genotype and overlapping clinic visits supported person-to-person transmission.	Multilocus genotyping plus transmission mapping	[120]
Genomic/deep and intermediate history	*Coccidioides immitis/C. posadasii*	Population genomics and admixture. Detected recent interspecific hybridization and lineage-specific differences in transposable-element control.	Whole-genome sequencing; reference-based SNPs; local ancestry and population-genetic analyses	[87,88]
Genomic/regional epidemiology	*Coccidioides* spp.	Western Hemisphere dispersal. Resolved local population structure, confirmed the two species and refined dispersal routes across the Americas.	Whole-genome reference-mapped SNPs; maximum-likelihood phylogeny; recombination analysis	[91,93,121]
Genomic/regional epidemiology	*Coccidioides posadasii*	Caribbean and northeastern South America. Identified a genetically differentiated Caribbean population and clarified its relationship to South American populations, including Brazil.	Whole-genome SNPs; maximum-likelihood phylogeny; population structure and demographic analyses	[90]
Genomic/outbreak and dispersal	*Cryptococcus gattii*	Pacific Northwest outbreak. Resolved multiple outbreak sublineages and connected Pacific Northwest genotypes to broader global diversity.	Whole-genome SNPs; maximum-likelihood phylogeny; geographic reconstruction	[95]
Genomic/outbreak and dispersal	*Cryptococcus gattii* VGII	Pacific Northwest outbreak. Showed that clonal outbreak groups arose from a recombining ancestral population followed by asexual expansion.	Whole-genome SNPs; population structure; recombination and linkage analyses	[122]
Genomic/global population scale	*Cryptococcus neoformans*	Global population structure and virulence. Resolved major lineages, extensive aneuploidy and candidate genomic correlates of virulence.	Whole-genome reference-mapped SNPs; maximum-likelihood phylogeny; GWAS and copy-number analysis	[96]
Genomic/within-host scale	*Cryptococcus neoformans*	Recurrent cryptococcal meningitis. Distinguished relapse from reinfection and identified within-host mutation, aneuploidy and potential adaptation during recurrent disease.	Serial-isolate whole-genome sequencing; SNP and copy-number comparisons	[123]
Genomic/global emergence	*Candida auris*	Three-continent emergence. Demonstrated near-simultaneous emergence of deeply separated geographic clades and clade-specific resistance mutations.	Whole-genome reference-mapped SNPs; maximum-parsimony/maximum-likelihood phylogeny; epidemiologic integration	[98]
Genomic/outbreak scale	*Candida auris*	United Kingdom hospital outbreak. Resolved persistent transmission within a cardiothoracic center and linked environmental and patient isolates.	Whole-genome SNPs; maximum-likelihood phylogeny; epidemiologic and environmental sampling	[99]
Genomic/global emergence	*Candida auris*	Global phylogeography and timing. Confirmed four widespread clades, estimated their recent expansions and showed that resistance mechanisms differed among clades.	Whole-genome SNPs; maximum-likelihood and Bayesian molecular-clock phylogenies; population genomics	[103]
Genomic/environmental acquisition	*Aspergillus fumigatus*	Clinical-environmental transmission and azole resistance. Demonstrated that closely related azole-resistant genotypes occur in patients and the environment, supporting environmental acquisition.	Whole-genome SNPs; maximum-likelihood phylogeny; population structure and resistance-genotype analysis	[104]
Genomic/outbreak scale	*Apophysomyces trapeziformis*	Tornado-associated mucormycosis. Showed that the post-tornado cluster involved several distinct strains rather than expansion of a single clone.	Whole-genome sequence typing; reference-free and SNP-distance comparisons	[105]
Genomic/species boundaries	*Histoplasma* species complex	Genome-scale species delimitation. Confirmed deeply separated cryptic species and showed that traditional taxonomic varieties did not capture genomic boundaries.	Whole-genome SNPs and ortholog phylogenies; concordance and population-genetic analyses	[124,125,126]
Genomic/host association	*Pneumocystis* species	Population history across mammalian hosts. Revealed host-associated species, low recombination and distinct demographic histories inconsistent with simple host–parasite codivergence.	Whole-genome and large-scale multilocus data; coalescent demographic and phylogenomic analyses	[127]
Genomic/zoonotic epidemic	*Sporothrix brasiliensis*	Brazilian cat-transmitted sporotrichosis. Resolved epidemic population structure, routes of geographic expansion and repeated introductions beyond the main southeastern focus.	Whole-genome SNPs; phylogenomics; population structure and phylogeographic analysis	[106]
Genomic/endemic population scale	*Talaromyces marneffei*	Vietnamese clinical population. Linked pathogen population structure and genetic variation to geographic and clinical phenotypes across an endemic region.	Whole-genome SNPs; maximum-likelihood phylogeny; population structure and genome-wide association	[107]
Genomic/outbreak scale	*Rhizopus* and *Mucor* spp.	Transplant-center mucormycosis cluster. Genome sequencing showed that temporally associated cases involved genetically distinct organisms, arguing against an outbreak caused by a single clone.	Whole-genome sequencing; pairwise genomic comparison integrated with epidemiology	[128]

Abbreviations: eBURST, based upon related sequence types, an algorithm for grouping related multilocus sequence types; GWAS, genome-wide association study; ITS, internal transcribed spacer; MLST, multilocus sequence typing; PCR, polymerase chain reaction; SNP, single-nucleotide polymorphism. VGII denotes molecular type II of the *Cryptococcus gattii* species complex. “spp.” denotes multiple species within a genus.

## Data Availability

The source data and code underlying Figure 1, Figure 2 and Figure 3 are available at https://doi.org/10.6084/m9.figshare.34719051.
